# Self-Help Plus for refugees and asylum seekers: an individual participant data meta-analysis

**DOI:** 10.1136/bmjment-2023-300672

**Published:** 2023-07-31

**Authors:** Eirini Karyotaki, Marit Sijbrandij, Marianna Purgato, Ceren Acarturk, Daniel Lakin, Della Bailey, Emily Peckham, Ersin Uygun, Federico Tedeschi, Johannes Wancata, Jura Augustinavicius, Kenneth Carswell, Maritta Välimäki, Mark van Ommeren, Markus Koesters, Mariana Popa, Marx Ronald Leku, Minna Anttila, Rachel Churchill, Ross G White, Sarah Al-Hashimi, Tella Lantta, Teresa Au, Thomas Klein, Wietse A Tol, Pim Cuijpers, Corrado Barbui

**Affiliations:** 1 Department of Clinical, Neuro-, and Developmental Psychology and WHO Collaborating Centre for Research and Dissemination of Psychological Interventions, Vrije Universiteit Amsterdam, Amsterdam, The Netherlands; 2 Amsterdam Public Health Research Institute, Amsterdam, The Netherlands; 3 WHO Collaborating Centre for Research and Training in Mental Health and Service Evaluation, Department of Neuroscience, Biomedicine, and Movement Sciences, Section of Psychiatry, University of Verona, Verona, Italy; 4 Department of Psychology, Koc University, Istanbul, Turkey; 5 Department of Mental Health, Bloomberg School of Public Health, Johns Hopkins University, Baltimore, Maryland, USA; 6 Department of Health Sciences, University of York, York, UK; 7 Department of Trauma and Disasters, Bilge University, Ankara, Turkey; 8 Clinical Division of Social Psychiatry, Department of Psychiatry and Psychotherapy, Medical University of Vienna, Vienna, Austria; 9 Department of Mental Health and Substance Use, World Health Organization, Geneva, Switzerland; 10 Department of Nursing Science, University of Turku, Turku, Finland; 11 Xiangya School of Nursing, The Xiangya Evidence-Based Practice and Healthcare Innovation, Central South University, Chang, People's Republic of China; 12 Department of Psychiatry II, Ulm University, Ulm, Germany; 13 School of Psychology, Queen's University Belfast, Belfast, UK; 14 HealthRight Uganda, Arua, Uganda; 15 Centre for Reviews and Dissemination, University of York, York, UK

**Keywords:** Depression & mood disorders, Adult psychiatry

## Abstract

**Question:**

Refugees and asylum seekers are at high risk of mental disorders due to various stressors before, during and after forceful displacement. The WHO Self-Help Plus (SH+) intervention was developed to manage psychological distress and a broad range of mental health symptoms in vulnerable populations. This study aimed to examine the effects and moderators of SH+ compared with Enhanced Care as Usual (ECAU) in reducing depressive symptoms among refugees and asylum seekers.

**Study selection and analysis:**

Three randomised trials were identified with 1795 individual participant data (IPD). We performed an IPD meta-analysis to estimate the effects of SH+, primarily on depressive symptoms and second on post-traumatic stress, well-being, self-identified problems and functioning. Effects were also estimated at 5–6 months postrandomisation (midterm).

**Findings:**

There was no evidence of a difference between SH+ and ECAU+ in reducing depressive symptoms at postintervention. However, SH+ had significantly larger effects among participants who were not employed (β=1.60, 95% CI 0.20 to 3.00) and had lower mental well-being levels (β=0.02, 95% CI 0.001 to 0.05). At midterm, SH+ was significantly more effective than ECAU in improving depressive symptoms (β=−1.13, 95% CI −1.99 to −0.26), self-identified problems (β=−1.56, 95% CI −2.54 to −0.59) and well-being (β=6.22, 95% CI 1.60 to 10.90).

**Conclusions:**

Although SH+ did not differ significantly from ECAU in reducing symptoms of depression at postintervention, it did present benefits for particularly vulnerable participants (ie, unemployed and with lower mental well-being levels), and benefits were also evident at midterm follow-up. These results are promising for the use of SH+ in the management of depressive symptoms and improvement of well-being and self-identified problems among refugees and asylum seekers.

**WHAT IS ALREADY KNOWN ON THIS TOPIC**

- Refugees and asylum seekers are at high risk of mental disorders like depression.
- Effective, low-intensity and scalable interventions are needed to meet the mental health needs of this disadvantaged population.
- Very little is known regarding individual participant differences in response to the WHO Self-Help Plus (SH+) intervention among refugees and asylum seekers.

**WHAT THIS STUDY ADDS**

- SH+ has promising effects in reducing depressive symptoms over the midterm and in improving self-identified problems and well-being.
- Employment and levels of well-being significantly moderated SH+ effects.

**HOW THIS STUDY MIGHT AFFECT RESEARCH, PRACTICE OR POLICY**

- The present work offers encouraging evidence supporting scaling up SH+ to address the mental health needs of refugees and asylum seekers.
- The identified moderators could inform SH+ tailoring, thereby enhancing its effectiveness in reducing symptoms of depression in refugees and asylum seekers.

## Introduction

In 2022, more than 101 million people were forcibly displaced worldwide because of war, armed conflict, persecution, violence and human rights violations.^1^ Before, during and after their forceful displacement, individuals face various stressors, including socioeconomic deprivation, discrimination, lack of social integration, poverty and unemployment.^2–5^ It has been found that approximately 22% of refugees and asylum seekers suffer from common mental disorders like depression.^6^ Yet accessing psychological care is often particularly difficult for refugees and asylum seekers due to barriers, including lack of services, language, lack of information about existing healthcare facilities in the host country, stigma and treatment-related costs.^7^ Scalable low-intensity psychological interventions may provide significant benefits in addressing the substantial unmet mental health needs of refugees and asylum seekers.

The WHO has developed a guided self-help intervention, named Self-Help Plus (SH+), to fill the gap between the limited treatment supply and enormous demand in humanitarian settings.^8^ SH+ uses a task-sharing approach (ie, the delegation of care tasks to non-specialist peer facilitators) to address a broad range of mental health problems^8^ in under-resourced settings. The first trials that examined this intervention have shown promising outcomes.^9–11^ However, it is unclear whether all individuals benefit from SH+. Identifying subgroups of individuals for whom this intervention may be more effective would greatly assist the targeted dissemination and implementation of SH+. Advanced meta-analytical approaches are required to get a precise estimate of SH+ effects and explore individual characteristics as effect modifiers. One of the most well-known methods is the individual participant data (IPD) meta-analysis approach, which uses raw data from randomised trials (RCTs), thereby improving overall estimates and maximising the power to identify participant characteristics that predict treatment outcomes.^12^

### Objective

We conducted an IPD meta-analysis to examine the effects of SH+ compared with Enhanced Care as Usual (ECAU) in reducing depressive symptoms among refugees and asylum seekers. Secondary outcomes were post-traumatic stress symptoms, well-being, self-identified problems and functioning. We also aimed to evaluate the moderating effects of participants’ sociodemographic, migratory and mental health characteristics on the differential effectiveness of SH+ relative to ECAU in reducing depressive symptoms among refugees and asylum seekers.

## Methods

The methods are described in detail in our published protocol^13^ and its registration (https://osf.io/jg4hs).

### Eligibility criteria and selection of studies

For the present IPD meta-analysis, a systematic literature search was not needed. We focused on the SH+ intervention, which was not publicly available when this study was conducted, and all trials on SH+ required to be approved by the WHO. Thus, through the WHO, we identified three RCTs^9–11^ examining the effects of SH+ on adult (≥18 years of age) refugees and asylum seekers with elevated levels of psychological distress based on self-report outcome measures (≥3 on the 12-item General Health Questionnaire (GHQ-12)^14^ or ≥5 on the Kessler-6 (K-6) Scale).^9^ In two trials, participants with mental disorders at baseline were excluded, based on the The Mini International Neuropsychiatric Interview (MINI). ‘Refugees and asylum seekers’ were defined as individuals who (A) Were recognised as having a refugee status under the 1951 United Nations Convention, (B) Sought international protection but whose application for refugee status had not yet been concluded, or (C) Were under temporary protection.^15^

Although a systematic literature search was not needed, to increase replicability and rule out any possibility of missing existent trials conducted after the intervention release, we ran a search strategy on 28 March 2023. We obtained 1461 records, with 15 full texts potentially eligible. No additional studies tested SH+ as a stand-alone intervention for asylum seekers and refugees (see online supplemental appendix A).

10.1136/bmjment-2023-300672.supp1 Supplementary data

### The SH+ intervention

Details about the intervention are summarised in our protocol.^13^ In brief, the WHO SH+ intervention comprises 5 weekly 1½-hour to 2-hour group sessions, like a class or a workshop. It is a transdiagnostic intervention, designed to help adults cope with stress and manage adversity. SH+ can be adapted for different cultures and languages and is suitable for people with general distress, which is common among many different psychological problems, whether they meet the criteria for a diagnosable mental disorder. It is based on Acceptance and Commitment Therapy (ACT), in which participants learn to accommodate difficult thoughts and feelings while finding ways to act according to their values. SH+ uses prerecorded audio and an illustrated self-help book to teach stress management skills (see https://www.who.int/publications/i/item/9789240003927). This format enables delivery by briefly trained non-specialist peer facilitators, who play audio, encourage participants to practise the guided stress management exercises, read out discussion questions to make the groups interactive, and address any questions or safety issues that may arise. In the studies used for the present IPD meta-analysis, facilitators were non-specialists and some experienced as volunteers, community workers or similar profiles, and some had worked in healthcare or related settings.^9–11^

### Enhanced care as usual

In this control group, participants received routine social support or healthcare support and treatment according to the local regulations and practices of the host country. The word ‘enhanced’ was used to emphasise that participants were provided with specific information and practical support on social, care, and legal services for asylum seekers and refugees available locally at each site.^9–11^

### Data collection and data items

Study-level variables were extracted from the published reports of the trials, including the time of postintervention assessment, the country where the study was conducted, the target group and data related to the risk-of-bias assessment as described below. Regarding individual participant-level variables, the primary authors of the trials provided data for each participant at baseline, post-treatment, and 5 to 6 month post-randomisation scores of depressive and post-traumatic stress symptoms, functioning, self-identified problems, and well-being. Next, all information related to sociodemographic and migration information was gathered and synthesised per participant: that is, the author provided sociodemographic information related to gender, age, country, relationship status, educational level, years of education, employment and length of stay in the host country. Finally, the authors provided data about exposure to traumatic events. Details about the procedures of data collection can be found in our published protocol.^13^

### Risk of bias assessment

The Cochrane risk of bias tool 2.0^16^ was used to assess the risk of bias in the included studies arising from randomisation, allocation concealment, deviations from intended interventions and outcome measurement. In this study, we did not evaluate bias arising from missing data because missing values were addressed in our analyses, and bias related to the selection of reported outcomes because we had access to the full primary databases of the trials. The risk of bias was performed using information available in the published reports of the studies. In case of unclear items, the primary authors were asked to provide clarifications.

### Outcomes

The primary outcomes of this IPD meta-analysis were postintervention symptoms of depression on the Participant Health Questionnaire 9-items (PHQ-9).^17^ Secondary outcomes were symptoms of post-traumatic stress disorder on the 6-item version of the post-traumatic stress disorder (PTSD) Checklist-civilian version (PCL-6)^18 19^ and improvements in functioning on WHO Disability Assessment Schedule (WHODAS 2.0),^20^ self-identified problems on Psychological Outcome Profiles (PSYCHLOPS),^21^ and well-being on WHO Well-Being Index (WHO-5).^22^ Further, we also examined all outcomes at 5–6 months postrandomisation.

### Data analysis

We performed the analyses under the intention-to-treat principle. Missing values were handled by multiple imputation under the missing-at-random assumption (20 imputations). To test the robustness of our findings, we performed sensitivity analyses using complete cases only.

In a one-stage IPD meta-analysis, we synthesised all data from all trials with participants clustered within trials. The clustering of participants is accounted for using a random intercept for each study. The one-stage IPD meta-analysis approach offers more sophisticated modelling of the moderators, and thus, it is preferred over the two-stage IPD approach.^23 24^ We calculated the standardised β coefficient for the comparison between SH+ and ECAU. This estimate indicates how many SDs the dependent variable (eg, depressive symptoms) changes per SD increase in the predictor variable. The higher the β is, the greater the effect of the predictor variable on the dependent variable, and there is no association among the variables if β is zero.

First, to examine the effects of SH+ on reduction in postintervention depressive symptoms (secondary outcomes and outcomes at 5–6 months postrandomisation were examined in the same way), we performed a mixed-effects linear regression with random intercepts model with each trial having a random effect and a fixed effect for the intervention and the severity of depressive symptoms. The postintervention depressive symptoms were used as the dependent variable, while the condition (SH+ vs ECAU) was the independent variable while adjusting for baseline depression symptom severity. Second, we tested whether sociodemographic characteristics, mental health characteristics and migration variables moderated the effects of SH+ at postintervention. To do so, we added the interaction between each moderator and SH+ effect on depressive symptoms into the mixed-effects linear regression model. Each potential moderator was added into separate bivariate models.

To test the robustness of our findings, the analysis of the main outcomes was repeated using complete cases and in a two-stage IPD meta-analysis in which the data are analysed separately in each study and then the estimates are combined to calculate the pooled effect sizes for all outcomes using the random-effects model. Further, statistical heterogeneity was assessed using I^2^, with values of 0% indicating no heterogeneity, 25% low heterogeneity, 50% moderate heterogeneity and 75% high heterogeneity.^25^ To give the full magnitude of heterogeneity, the 95% CIs around I^2^ were calculated using the non-central χ^2^-based approach.^26^ Finally, publication bias was not applicable in this work because we had access to all trials using the WHO SH+ intervention. All analyses were conducted in STATA V.16.0.

To evaluate the certainty of the evidence of our primary outcome (ie, depressive symptom severity at postintervention), we used the Grading of Recommendations, Assessment, Development, and Evaluations (GRADE) methodology.^27^

## Results

### Study characteristics

Three RCTs were included in the present IPD meta-analysis with a total of 1795 participants comparing SH+ (n=883) to ECAU (n=912). These studies were conducted in western Europe (Italy, Austria, Finland, Germany and the UK),^10^ Turkey^11^ and Uganda.^9^ The included studies recruited adult (≥18 years of age) refugees and asylum seekers with elevated psychological distress levels based on cut-off scores ≥3^10 11^ on the GHQ or ≥5 on the K-6.^9^ Two RCTs included both genders,^10 11^ whereas one included only female participants.^9^ Two RCTs excluded individuals meeting criteria for a Diagnostic and Statistical Manual of Mental Disorders (DSM-5) diagnosis of mental disorder using the MINI Neuropsychiatric Interview^10 11^ and individuals with an acute medical condition.^10 11^ Further, imminent risk of suicide was an exclusion criterion in all three RCTs.

### Participant characteristics

Table 1 shows the participant characteristics at baseline. Most participants were female (n=1232/1795; 69%) married/cohabiting (n=1157/1777; 65%) with a mean age of 31.5 (SD=10) years. Approximately, half of the participants had been in primary school/junior high school (997/1766; 55%) and around 19% of participants were employed (n=337/1777). Most of the participants were from Syria (757/1795; 42%) and South Sudan (694/1795; 39%) and had spent a mean of 30 months (SD=33) in the host country. The mean (SD) baseline scores on the outcomes of interest were the following: 10.2 (6.3) on PHQ-9; 10.6 (6.5) on PCL-6; 0.27 (0.24) on WHODAS 2.0; 39.3 (24) on WHO-5; and 13.3 (5.4) on PSYCHLOPS. These scores at postintervention were: 8.3 (6.1) on PHQ-9; 11.1 (7.9) on PCL-6; 0.25 (0.67) on WHODAS 2.0, 47.1 (24) on WHO-5; and 10.5 (6.04) on PSYCHLOPS. Finally, the mean (SD) scores on the outcomes of interest at 5–6 months postrandomisation were: 7.8 (5.7) on PHQ-9; 15.9 (10.7) on PCL-6; 0.24 (0.23) on WHODAS 2.0, 48.5 (23.4) on WHO-5; and 9.3 (6.1) on PSYCHLOPS.

**Table 1:** Participant characteristics at baseline

Means	Total sample	SH+	ECAU
	M (SD), n	M (SD), n	M (SD), n
Age (years)	31.48 (10.07), 1779	31.55 (9.99), 870	31.41 (10.16), 909
Length of stay (months)	29.77 (33.01), 1308	32.03 (36.98), 680	27.32 (27.90), 628
PHQ-9	10.23 (6.35), 1741	10.19 (6.35), 852	10.28 (6.36), 889
PCL-6	10.67 (6.53), 1744	10.69 (6.49), 855	10.65 (6.56), 889
WHODAS 2.0	.27 (.24), 1721	.27 (.24), 838	.27 (.24), 883
PSYCHLOPS	13.32 (5.39), 1561	13.36 (5.30), 761	13.28 (5.48), 800
WHO-5	39.29 (24.03), 1770	38.70 (23.99), 864	39.85 (24.07), 906

Frequencies	n/ n total (%)	n/ n total (%)	n/ n total (%)
Gender (female)	1232/1795 (31.36)	602/883 (68.18)	630/912 (69.08)
Country of origin			
Nigeria	114/1795 (6.35)	59/883 (6.68)	55/912 (6.03)
Syria	757/1795 (42.17)	381/883 (43.15)	376/912 (41.23)
Iraq	94/1795 (5.24)	44/883 (4.98)	50/912 (5.48)
South Sudan	694/1795 (38.66)	331/883 (37.49)	363/912 (39.80)
Other	136/1795 (7.58)	68/883 (7.70)	68/912 (7.46)
In a romantic relationship	1157/1777 (65.11)	563/870 (64.71)	594/907 (65.49)
Education level			
Illiterate	270/1766 (15.29)	128/863 (14.83)	142/903 (15.73)
Primary school/junior high school	977/1766 (55.32)	483/863 (55.97)	494/903 (54.71)
High school	324/1766 (18.35)	154/863 (17.84)	170/903 (18.83)
University degree and above	195/1766 (11.04)	98/863 (11.36)	97/903 (10.74)
(Self-)employed	337/1777 (18.96)	169/869 (19.45)	168/908 (18.50)
Traumatic experiences			
Lack of food or water	1178/1775 (66.37)	572/870 (65.75)	606/905 (66.96)
No medical access	909/1775 (51.21)	433/870 (49.77)	476/905 (52.60)
Lack of shelter	1048/1773 (59.11)	509/868 (58.64)	539/905 (59.56)
Imprisonment	379/1772 (21.39)	191/870 (21.95)	188/902 (20.84)
Serious injury	533/1773 (30.06)	264/868 (30.41)	269/905 (29.72)
Combat	847/1774 (47.75)	429/870 (49.31)	418/904 (46.24)
Rape or sexual abuse	240/1771 (13.55)	111/867 (12.80)	129/904 (14.27)
Close to death	861/1773 (48.56)	422/868 (48.62)	439/905 (48.51)
Murder	933/1778 (52.47)	454/870 (52.18)	479/908 (52.75)
Abduction	413/1772 (23.31)	212/867 (24.45)	201/905 (22.21)
Torture	808/1773 (45.57)	395/869 (45.45)	413/904 (45.69)

ECAU, Enhanced Care as Usual; n, number of participants; PCL-6, Post-Traumatic Stress Disorder Symptom Checklist – five items; PHQ-9, Patient health Questionnaire – 9 items; PSYCHLOPS, Psychological Outcome Profiles; SH+, Self-Help Plus; WHO-5, The WHO Five Well-Being Index; WHODAS 2.0, WHO Disability Assessment Schedule.

### Risk of bias assessment

All included studies were at low risk of bias across most domains. All trials had adequate randomisation and allocation concealment, and did not deviate from the intended intervention since all interventions were administered per protocol using an illustrated self-help book and prerecorded audio guide. Further, the non-specialist peer facilitators had adequate training and regular supervision to ensure intervention fidelity. Missing data were handled by the present Individual Participant Data Meta-Analysis (IPDMA) using multiple imputation. The percentage of missing values was small across the included studies (ie, 16.1% (289/1795) missingness at postintervention). Further, the missing values were acceptably balanced across the SH+ (17.5%) and ECAU (13.1%) conditions. Although all studies used assessors masked to the outcome of randomisation, participants were not masked due to the nature of the SH+ intervention; thus, we cannot rule out bias in the measurement of the outcome across all the included studies.

### Effects of SH+ on depressive symptoms

**Figure 1:**
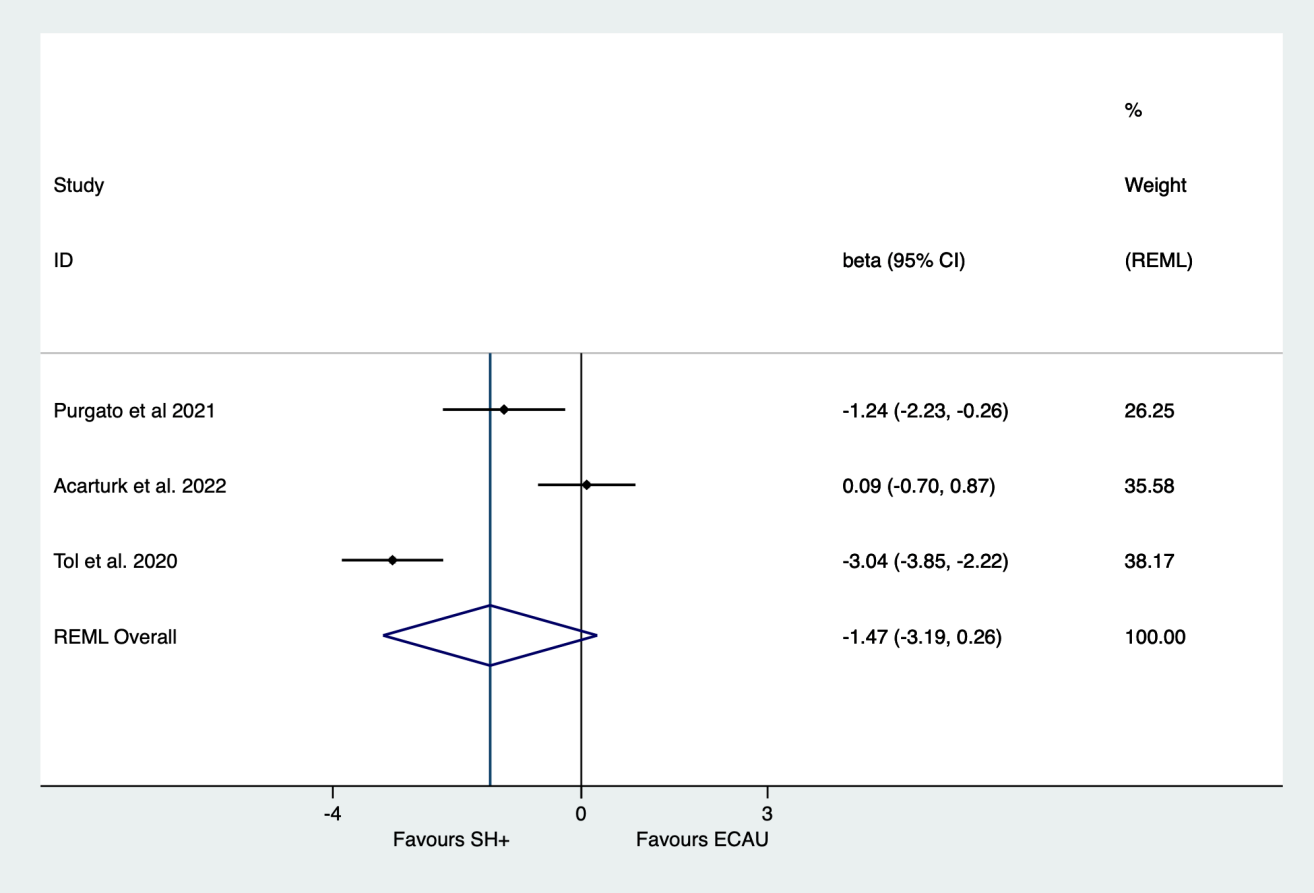
Main effects of SH+ compared to ECAU in reducing symptoms of depression and postintervention. REML, restricted maximum likelihood.

Table 2 presents the main outcomes of one-stage IPDMA on depressive symptoms (results of two-stage IPDMA are presented in online supplemental table a in the Appendix). At postintervention, we found an effect of β=−1.47 (95% CI −3.19 to 0.26) in favour of SH+ compared with ECAU in reducing depressive symptoms at post-test, which failed to reach statistical significance (p=0.09; see figure 1). According to the GRADE assessment, the strength of this evidence is evaluated as moderate (online supplemental table b). Similar results were observed in complete case and two-stage IPDMAs. Heterogeneity was high (I^2^=92%; 95% CI 80% to 97%). In contrast with postintervention, at 5–6 months postrandomisation, the results of the one-stage IPDMA indicated a significant effect in favour of SH+ compared with ECAU in reducing symptoms of depression (β=−1.13; 95% CI −1.99 to −0.26, p=0.01). Similar results were observed in the complete case (β=−1.41, 95% CI −2.19 to −0.64, p=0.000) and two-stage IPDMAs (β=−1.41, 95% CI −2.19 to −0.63, p=0.000). Heterogeneity dropped to 49% (95% CI 0% to 85%).

10.1136/bmjment-2023-300672.supp2 Supplementary data

10.1136/bmjment-2023-300672.supp3 Supplementary data

**Table 2:** Effects of SH+ compared with ECAU on primary and secondary outcomes

Outcome	Imputed sample β	95% CI	P	Complete cases β	95% CI	P value
Postintervention						
Main outcome						
PHQ-9	−1.47	−3.19 to 0.26	0.09	−1.67	−3.43 to 0.08	0.06
Secondary outcomes						
PCL-6	−1.11	−3.10 to 0.89	0.28	−1.24	−3.33 to 0.83	0.24
WHODAS 2.0	−0.03	−0.10 to 0.04	0.35	−0.03	−1.08 to 0.04	0.34
PSYCHLOPS	−1.56	−2.54 to −0.59	0.001	−1.95	−2.69 to −1.21	0.000
WHO-5	6.22	1.60 to 10.90	0.009	−7.39	2.85 to 11.9	0.001
5–6 months postrandomisation						
Main outcome						
PHQ-9	−1.13	−1.99 to −0.26	0.01	−1.41	−2.19 to −0.64	0.000
Secondary outcomes						
PCL-6	−0.72	−1.58 to −0.13	0.10	−0.85	−1.71 to −0.20	0.06
WHODAS 2.0	−0.01	−0.03 to 0.02	0.49	0.01	−0.037 to .017	0.46
PSYCHLOPS	−0.78	−1.53 to 0.04	0.04	−0.96	−1.62 to −0.31	0.004
WHO-5	4.15	1.44 to 6.87	0.003	5.10	2.10 to 8.10	0.001

ECAU, Enhanced Care as Usual; PCL-6, Post-Traumatic Stress Disorder Symptom Checklist – five items; PHQ-9, Patient health Questionnaire – 9 items; PSYCHLOPS, Psychological Outcome Profiles; SH+, Self-Help Plus; WHO5, The WHO Five Well-Being Index; WHODAS 2.0, WHO Disability Assessment Schedule.

### Effects of SH+ on secondary outcomes

There was no evidence of a difference between SH+ and ECAU in reducing symptoms of post-traumatic stress and improving functioning at postintervention and 5–6 months postrandomisation. These results were confirmed by the complete case and two-stage IPDMAs. However, the SH+ outperformed ECAU in improving self-identified problems (β=−1.56, 95% CI −2.54 to −0.59; p=0.001) and mental well-being (β=6.22, 95% CI 1.60 to 10.90, p=0.009) at post-test. Results were replicated in complete case (self-identified problems: β=−1.95, 95% CI −2.69 to −1.21, p=0.000; mental well-being: β=−7.39, 95% CI 2.85 to 11.9; p=0.001) and two-stage IPDMAs (self-identified problems: β=−1.95, 95% CI −2.68 to −1.22, p=0.000; mental well-being: β=7.39, 95% CI 2.72 to 12.0, p=0.002). Heterogeneity was 37% (95% CI 0% to 80%) for self-identified problems and 72% (95% CI 7% to 92%) for mental well-being. Similarly, at 5–6 months postrandomisation, there was a significant small effect in favour of SH+ on self-identified problems (β=−0.78, 95% CI −1.53 to .04, p=0.04), which was replicated in complete case analysis (β=−0.96; 95% CI −1.62 to −0.31, p=0.004) and two-stage IPDMAs (β=−0.96, 95% CI −1.68 to −0.24, p=0.009). Heterogeneity was 31% (95% CI 0% to 93%). Finally, SH+ outperformed ECAU in improving mental well-being (β=4.15, 95% CI 1.44 to 6.87, p=0.003) at 5–6 months postrandomisation. These results were replicated in complete case (β=5.10, 95% CI 2.10 to 8.10, p=0.001) and two-stage IPDMAs (β=5.09, 95% CI 2.16 to 8.02, p=0.001). Heterogeneity was 32% (95% CI 0% to 93%). All secondary outcomes are presented in table 2 and online supplemental table a.

### Moderators of the effects of SH+ versus ECAU on depressive symptoms at postintervention

The results of the moderator analyses showed that employment was associated with reduction of depressive symptoms at postintervention (β=1.60, 95% 0.20 to 3.01, p=0.02), suggesting that those who were not employed benefited more from SH+ compared with ECAU. The moderating effect of employment was replicated in complete case analysis (β=0.1.81, 95% CI 0.44 to 3.20, p=0.01). Further, the lower the mental well-being at the baseline the greater the effects of SH+ compared with ECAU in reducing depressive symptoms at postintervention (β=0.02, 95% CI 0.001 to 0.05, p=0.04). The moderating effect of mental well-being was confirmed in complete case analysis (β=0.03, 95% 0.01 to 0.05, p=0.01). None of the other examined variables was significantly associated with SH+ outcomes (see online supplemental table c and d for complete case analyses).

10.1136/bmjment-2023-300672.supp4 Supplementary data

10.1136/bmjment-2023-300672.supp5 Supplementary data

## Discussion

In this study we performed an IPD meta-analysis to examine the overall effects and participant-level moderators of SH+ compared with ECAU in reducing depressive symptoms among refugees and asylum seekers. Further, we aimed to investigate the SH+ impact on post-traumatic stress symptoms, functioning, self-identified problems and well-being. We found no evidence of a difference between SH+ and ECAU in reducing depressive symptoms at postintervention. Yet, we found that participants who were unemployed and those who had lower well-being at baseline benefited more from SH+ than ECAU. Importantly, over the midterm (ie, 5–6 months postrandomisation), SH+ was more effective than ECAU in reducing depressive symptoms. Finally, regarding secondary outcomes, SH+ was more effective than ECAU in improving self-identified problems and well-being at both postintervention and follow-up, whereas there was no evidence of a difference between SH+ and ECAU in reducing post-traumatic stress symptoms and improving functioning at both time points.

The finding that SH+ did not have immediate effects on depressive and post-traumatic stress symptoms is not in line with the results of recent meta-analyses on the efficacy of higher intensity psychological interventions in refugees and asylum seekers.^28 29^ Turrini and colleagues and Kip and colleagues found large effects in favour of psychological interventions compared with controls in decreasing symptoms of depression (standardised mean difference (SMD)=0.82–1.02) and post-traumatic stress (SMD=0.71–0.77) at post-test.^28 29^ Still, the heterogeneity among the examined trials was large (I^2^=83%–89%), suggesting great variability among the effects of psychological interventions.^28^ We should note that these previous meta-analytical findings have mainly been drawn from studies focusing on relatively higher-intensity treatments, probably more tailored to individual patient needs, delivered by skilled therapists.^28 29^ Next, most of the participants in those studies^28 29^ had mental disorders such as depression. It is, therefore, reasonable to expect a greater effect of psychological interventions versus controls on those who experience mental disorders rather than elevated levels of distress.

In contrast with postintervention, we did observe a significant impact on symptoms of depression at midterm. A plausible explanation for the change in the effects on depressive symptoms between postintervention and midterm may be the ‘incubation’ period needed for fully realising the impact of ACT.^30–33^ Further, two of the included studies focused on prevention.^10 11^ Thus, participants had mild symptoms of depression at the baseline and probably little room for differences in symptoms at the postintervention assessment. The present findings on self-identified problems and well-being replicated the results of previous meta-analyses showing small-to-moderate effects of ACT on these outcomes.^34^ Such positive short-term and midterm impact is very much in line with the main goal of ACT, which is not the reduction in symptoms of mental disorders, but the promotion of well-being.^34 35^

To our knowledge, this is the first study examining moderators of psychological interventions for refugees and asylum seekers at an IPD meta-analysis level. We found that SH+ was more effective for unemployed participants and those who had lower levels of well-being at baseline. Unemployment/underemployment has long been listed among the most critical resettlement-related stress sources.^36 37^ Thus, the greater effects of SH+ among participants who were not employed may be partly related to the initial severity of their mental health-related and life-related problems. Further, given that ACT primarily targets well-being, as discussed above, it is not surprising that this intervention works better among individuals with impaired well-being.

The present findings, nonetheless, should be interpreted considering several limitations. First, in our primary analyses (ie, depression outcomes at postintervention), we observed considerable and significant heterogeneity, suggesting that the effects of SH+ varied among the included studies. Although the actual reason for this heterogeneity remains unclear, a possible reason for this discrepancy in studies’ effects is that refugees might have faced different postmigration stressors in the countries where the studies were conducted. Another plausible explanation is the difference in the eligibility criteria among the included studies, that is, two studies focused on prevention which included only participants without a mental disorder at baseline. The study in Uganda did not have this exclusion criterion and included both people with moderate and severe psychological distress according to the K-6 and did not perform diagnostic interviews. It therefore possibly included participants who met a diagnosis of depression. Second, we should also be mindful of the clinical diversity of the present sample. Although we examined a wide range of possible individual-level moderators to understand the clinical heterogeneity, there are important variables that we could not investigate in the present analyses (eg, history of mental health problems, duration of symptoms, etc). Future studies should explore such factors to better understand the effectiveness of SH+. Third, only three RCTs were conducted on this topic, suggesting that our conclusions are limited because of the small number of existing trials. Nevertheless, the number of participants was large, justifying the use of the IPD meta-analysis methodology. Fourth, we should acknowledge that the effect of SH+ in this study on self-identified problems and well-being might not be generalisable to interventions on asylum seekers and refugees affected by mental disorders since the present sample predominately consists of individuals with elevated levels of distress. Finally, the present analysis could not examine longer-term data since only one trial reported such outcomes. Future research should examine the effects of SH+ in the longer term.

Informed by the finding of the positive midterm effects, WHO has made SH+ publicly available (see https://www.who.int/publications/i/item/9789240035119), and the present work provides essential insights to facilitate SH+ targeted dissemination. Considering the beneficial midterm outcomes, SH+ appears to be an appropriate intervention for the management of depressive symptoms among refugees and asylum seekers. Based on the present outcomes, SH+ can also be seen as a strategy to help the most vulnerable refugees and asylum seekers who struggle with unemployment and impaired well-being. Given its large group format, it may further be a useful first-line intervention to support refugees and asylum seekers and combined with more intensive interventions should they be required. SH+ could serve to boost psychological resilience through successful behavioural adaptation. Future studies should investigate adherence rates of SH+ and provide insights into whether increased well-being leads to better integration of refugees and asylum seekers in the host countries.

In conclusion, although SH+ did not significantly reduce depressive symptoms at postintervention, the present findings are important considering the improvements in depressive symptoms and substantial benefits in well-being and self-identified problems. Next to that, subgroups of refugees and asylum seekers appear to benefit more from this intervention. Considering these benefits, SH+ could be scaled up as a public health strategy to improve well-being, self-identified problems, and possibly address depressive symptoms among refugees and asylum seekers.

## Data Availability

Data are available upon reasonable request. A data-access request should be addressed to CB. Data can be shared upon the approval of the DEFining and Implementing Novel Evidence-based psychosocial interventions (RE-DEFINE) consortium.
