# Supplementary material for: Self-Help Plus for refugees and asylum seekers: an individual participant data meta-analysis
Source: BMJ Ment Health. 2023 Jul 31;26(1):e300672. doi: 10.1136/bmjment-2023-300672 (PMC10391800; doi:10.1136/bmjment-2023-300672)
Supplement: Supplementary data [file bmjment-2023-300672supp001.pdf]

Appendix A

**Search strings PubMed - March 28th, 2023**
**("world health organization"[MeSH Terms] OR "world health organization"[Title/Abstract]) AND ("psychosocial intervention"[MeSH Terms] OR "psychosocial intervention*"[Title/Abstract] OR "psychological intervention*"[Title/Abstract] OR "self help plus"[Title/Abstract] OR "sh"[Title/Abstract] OR "problem management plus"[Title/Abstract] OR "group problem management"[Title/Abstract] OR "pm"[Title/Abstract] OR "early adolescent skills"[Title/Abstract] OR "EASE"[Title/Abstract] OR ("doing"[Title/Abstract] AND "what matters"[Title/Abstract]) OR "DWM"[Title/Abstract] OR "step by step"[Title/Abstract] OR "group interpersonal"[Title/Abstract] OR "thinking healthy"[Title/Abstract])**
**"World Health Organization" AND ("psychological intervention" OR "psychosocial intervention" OR "self help plus" OR "problem management plus" OR "group problem management" OR "early adolescent skills" OR ("doing" AND "what matters") OR "step by step" OR "group interpersonal" OR "thinking healthy" OR "SH" OR "PM" OR "DMW" OR "EASE")**
**"World Health Organization" AND ("psychological intervention" OR "psychosocial intervention" OR "self help plus" OR "problem management plus" OR "group problem management" OR "early adolescent skills" OR "doing what matters")**
**"World Health Organization" AND ("step by step" OR "group interpersonal" OR "thinking healthy")**
**"World Health Organization" AND ("SH+" OR "PM+" OR "DMW" OR "EASE")**

Below we report a summary of the identified studies, with reasons for those excluded:

- **Acarturk 2022** «Doing What Matters in Times of Stress” to Decrease Psychological Distress During COVID-19: A Randomised Controlled Pilot Trial» → WRONG INTERVENTION
- **Greene 2021** «Feasibility of Community Psychosocial Intervention for Women Doing What Matters (DWM)» → WRONG INTERVENTION
- **Kalra 2021** «Addressing Stress Amongst Female Entrepreneurs in Addis Ababa, Ethiopia: a Pilot Study» → WRONG INTERVENTION
- **Karekla 2022** «Acceptance and Commitment Group Therapy for Unaccompanied Minors» → WRONG INTERVENTION/WRONG POPULATION GROUP
- **Lorenzo-Luaces 2022** «Low-intensity Stepped Care for Internalizing Distress» → WRONG INTERVENTION
- **Lorenzo-Luaces 2023** «Acceptability and Outcomes of Transdiagnostic Guided Self-help Bibliotherapy for Internalizing Disorder Symptoms in Adults: A Fully Remote Nationwide Open Trial» → WRONG INTERVENTION
- **Acarturk 2022** «Effectiveness of a WHO self-help psychological intervention for preventing mental disorders among Syrian refugees in Turkey: a randomized controlled trial» → INCLUDED.
- **Karyotaki 2021** «Self-help plus for refugees and asylum seekers; study protocol for a series of individual participant data meta-analyses Self-Help Plus (SH+)»→ WRONG STUDY DESIGN

- **Luo 2022** «Psychological stress self-help interventions for healthcare workers in the context of COVID-19 in China: a randomized controlled trial protocol Self-Help Plus (SH+) → WRONG POPULATION GROUP
- **Park 2022** «Cost-effectiveness of the Self-Help Plus Intervention for Adult Syrian Refugees Hosted in Turkey» → SAME RCT OF ACARTURK 2022
- **Purgato 2021** «Effectiveness of Self-Help Plus in Preventing Mental Disorders in Refugees and Asylum Seekers in Western Europe: A Multinational Randomized Controlled Trial → INCLUDED
- **Riello 2021** «Effectiveness of self-help plus (SH+) in reducing anxiety and post-traumatic symptomatology among care home workers during the COVID-19 pandemic: a randomized controlled trial Self-Help Plus (SH+) → WRONG POPULATION GROUP
- **Tol 2020** «Guided self-help to reduce psychological distress in South Sudanese female refugees in Uganda: a cluster randomised trial Self-Help Plus (SH+)» → INCLUDED
- **Turrini 2022** «Long-term effectiveness of Self-Help Plus in refugees and asylum seekers resettled in Western Europe: 12-month outcomes of a randomised controlled trial» → SAME RCT OF PURGATO ER AL 2021
- **Bryant 2021** «Effect of a Stepped Care Intervention on Anxiety and Depression In Distressed People in Jordan» → WRONG INTERVENTION (STEPPED-CARE)
