# Supplementary material for: Self-Help Plus for refugees and asylum seekers: an individual participant data meta-analysis
Source: BMJ Ment Health. 2023 Jul 31;26(1):e300672. doi: 10.1136/bmjment-2023-300672 (PMC10391800; doi:10.1136/bmjment-2023-300672)
Supplement: Supplementary data [file bmjment-2023-300672supp002.pdf]

Table a. Effects of SH+ compared to ECAU on primary and secondary outcomes – 2 stage IPDMA

Outcome	2-stage IPDMA β	95%CI	p	I^2% (95%CI)	Tau^2
Post-intervention					
Main outcome					
PHQ-9	-1.67	-3.57 to 0.22	.8	92 (80 to 97)	2.56
Secondary outcomes					
PCL-6	-1.25	-3.51 to 1.01	.24	95 (89 to 98)	3.78
WHODAS	-.03	-1.0 to .033	.31	95 (90 to 98)	0.003
PSYCHLOPS	-1.95	-2.68 to -1.22	.000	37 (0 to 80)	0.16
WHO5	7.39	2.72 to 12.0	.002	72 (7 to 92)	12.2
5-6m post-randomisation					
Main outcome					
PHQ-9	-1.41	-2.19 to -.63	.000	49 (0 to 86)	0.23
Secondary outcomes					
PCL-5	-.85	-1.74 to .04	.06	67 (0 to 90)	0.41
WHODAS	-.01	-.03 to .015	.44	74 (14 to 92)	0.0004
PSYCHLOPS	-.96	-1.68 to -0.24	.009	31 (0 to 93)	0.13
WHO5	5.09	2.16 to 8.02	.001	32 (0 to 93)	2.19

Abbreviations: 95%CI = 95% Confidence Intervals; ECAU = Enhanced Care as Usual; IPDMA = Individual Patient Data Meta-analysis; m = month; p = p-value; PCL-6 = Post-Traumatic Stress Disorder Symptom Checklist – 5 items; PHQ-9 = Patient health Questionnaire – 9 items; PSYCHLOPS = Psychological Outcomes Profiles; SH+: Self-help plus; WHO5 = The World Health Organisation- Five Well-Being Index; WHODAS = World Health Organization Disability Assessment Schedule; β = beta coefficient
