# Supplementary material for: Self-Help Plus for refugees and asylum seekers: an individual participant data meta-analysis
Source: BMJ Ment Health. 2023 Jul 31;26(1):e300672. doi: 10.1136/bmjment-2023-300672 (PMC10391800; doi:10.1136/bmjment-2023-300672)
Supplement: Supplementary data [file bmjment-2023-300672supp003.pdf]

Question: SH+ compared to ECAU for depressive symptoms among refugees and asylum seekers
Setting: humanitarian setting

Certainty assessment							№ of patients		Effect		Certainty	Importance
№ of studies	Study design	Risk of bias	Inconsistency	Indirectness	Imprecision	Other considerations	SH+	ECAU	Relative (95% CI)	Absolute (95% CI)		
**Depressive symptoms at post-intervention (assessed with: PHQ-9)**												
3	randomised trials	not serious	serious[a]	not serious	not serious	none	883	912	β = -1.47 (-3.19, 0.26)	-	⨁⨁⨁◯ Moderate	CRITICAL

CI: confidence interval

Explanations

a. High heterogeneity
