# Supplementary material for: Self-Help Plus for refugees and asylum seekers: an individual participant data meta-analysis
Source: BMJ Ment Health. 2023 Jul 31;26(1):e300672. doi: 10.1136/bmjment-2023-300672 (PMC10391800; doi:10.1136/bmjment-2023-300672)
Supplement: Supplementary data [file bmjment-2023-300672supp004.pdf]

Table c. *Moderators of SH+ effects on symptoms depression at post-intervention – Imputed sample*

Moderators	***Nobs***	***Coef.***	***SE***	***95%CI***	***p***
Gender	1737				
Group		-.88	.92	-2.7 to .94	.34
Gender (female)*Group		-.87	.73	-2.3 to .56	.23
Age	1737				
Group		-1.39	1.25	-3.9 to 1.07	.27
Age*Group		.001	.028	-.05 to ..53	.97
Country	1074				
Group		1.35	.91	-3.14 to .45	.14
Country*Group		.43	.47	-.49 to 1.3	.36
Nigeria*Group		-.87	1.40	-3.6 to 1.9	.54
Syria*Group		.63	1.16	-1.65 to 2.91	.59
Iraq*Group		1.17	1.60	-1.97 to 4.30	.46
Relationship Status	1735				
Group		-1.74	.96	-3.64 to .15	.07
In a relationship*Group		.48	.61	-.71 to 1.67	.43
Educational Level	1726				
Group		-1.36	1.17	-3.66 to .94	.25
Educational level*Group		-.22	.36	-.93 to .48	.54
Primary school/junior high school*Group		.14	.80	-1.43 to 1.71	.92
High school*Group		-.10	1.00	-2.06 to 1.86	.92
University degree and above*Group		-.60	1.20	-2.95 to 1.76	.62
Employement	1735				
Group		-1.74	.89	-3.50 to -.01	.05
Employed*Group		1.60	.71	.20 to 3.00	.02**
Length of stay	1279				
Group		-1.37	.81	-2.98 to .22	.09
Length of stay*Group		.003	.01	-.02 to .03	.81

Post-traumatic symptoms at BL	1703				
Group		-1.01	.96	-2.90 to .86	.29
Post-traumatic symptoms at BL*Group		-.05	.05	-.15 to .06	.38
Depressive symptoms at BL	1737				
Group		-1.09	.99	-3.03 to .85	.27
Depressive symptoms at BL*Group		-.03	.05	-.14 to .07	.52
Traumatic experiences					
Lack of food or water	1735				
Group		-1.51	1.03	-3.54 to .52	.14
Lack of food or water*Group		.14	.66	-1.16 to 1.44	.83
No Medical Access	1735				
Group		-1.36	.95	-3.22 to .49	.15
No Medical Access*Group		-.11	.59	-1.26 to 1.05	.85
Lack of Shelter	1733				
Group		-1.45	1.02	-3.44 to .54	.15
Lack of Shelter*Group		.06	.66	-1.24 to 1.36	.92
Imprisonment	1732				
Group		-1.52	.93	-3.34 to .29	.10
Imprisonment *Group		.37	.70	-1.0 to 1.7	.60
Serious Injury	1733				
Group		-1.78	.96	-3.65 to .10	.06
Serious Injury*Group		1.06	.59	-0.10 to 2.23	.07
Combat	1734				
Group		-1.95	.78	-3.48 to -.42	.001
Combat*Group		1.03	.70	-.34 to 2.40	.14
Rape or Sexual Abuse	1731				

Group		-1.30	.88	-3.01 to .42	.14
Rabe or Sexual Abuse*Group		-.89	.79	-2.44 to .66	.26
Close to death	1733				
Group		-1.17	.96	-3.05 to .71	.22
Close to death*Group		-.52	.56	-1.62 to .57	.35
Murder	1737				
Group		-1.47	.96	-3.35 to .40	.12
Murder*Group		.09	.55	-.99 to 1.17	.86
Abduction	1733				
Group		-1.30	.96	-3.18 to .58	.17
Abduction*Group		-.57	.68	-1.91 to .76	.40
Torture	1733				
Group		-1.30	.91	-3.10 to .49	.15
Torture*Group		-.29	.60	-1.48 to .89	.62
Wellbeing	1729				
Group		-2.43	.95	-4.29 to -.57	.01
Wellbeing*Group		.02	.01	.001 to .05	.04**
Self-identified problems	1528				
Group		-1.41	1.20	-3.75 to .94	.24
Self-identified problems*Group		-.01	.06	-.13 to .12	.89
Functioning	1682				
Group		-.80	.80	-2.37 to .77	.32
Functioning*Group		-2.20	1.66	-5.47 to 1.06	.19
Distress levels	1731				
Group		-1.45	.86	-3.14 to .24	.09
Distress levels*Group		.02	.27	-.50 to .54	.94

Abbreviations: BL: Baselin; CI: Confidence Intervals; Coef: Coefficient; Nobs: Number of observations; p: p-value
