# Supplementary material for: Self-Help Plus for refugees and asylum seekers: an individual participant data meta-analysis
Source: BMJ Ment Health. 2023 Jul 31;26(1):e300672. doi: 10.1136/bmjment-2023-300672 (PMC10391800; doi:10.1136/bmjment-2023-300672)
Supplement: Supplementary data [file bmjment-2023-300672supp005.pdf]

Table d. *Moderators of SH+ effects on symptoms depression at post-intervention – Complete Cases*

Moderators	*Nobs*	*Coef.*	*SE*	*95%CI*	*p*
Gender	1440				
Group		-1.06	.94	-2.9 to .78	.26
Gender (female)*Group		-.96	.73	-2.4 to .47	.19
Age	1440				
Group		-1.60	1.23	-4.01 to .81	.19
Age*Group		-.002	.02	-.05 to .05	.93
Country	810				
Group		-1.74	.85	-3.41 to -.07	.04
Country*Group		-.61	.45	-.28 to 1.5	.18
Nigeria*Group		-.86	1.43	-3.7 to 1.96	.55
Syria*Group		.86	1.07	-1.2 to 2.96	.42
Iraq*Group		1.49	1.65	-1.7 to 4.72	.36
Relationship Status	1438				
Group		-2.08	.94	-3.9 to -.23	.03
In a relationship*Group		.59	.60	-.58 to 1.77	.32
Educational Level	1429				
Group		-1.25	1.10	-3.35 to .86	.24
Educational level*Group		-.28	.35	-.96 to .41	.42
Primary school/junior high school*Group		.07	.80	-1.50 to 1.62	.93
High school*Group		-.22	.97	-2.14 to 1.70	.82
University degree and above*Group		-.84	1.17	-3.13 to 1.45	.47
Employement	1438				
Group		-2.00	.90	-3.77 to -.23	.02
Employed*Group		1.81	.70	.44 to 3.20	.01**
Length of stay	1051				
Group		-1.70	.73	-3.14 to -.25	.02
Length of stay*Group		.004	.01	-.02 to .03	.72
Post-traumatic symptoms at BL	1414				
Group		-1.21	.93	-3.03 to .61	.20
Post-traumatic symptoms at BL*Group		-.05	.05	-.15 to .05	.32
Depressive symptoms at BL	1440				
Group		-1.18	.95	-3.05 to .67	.21
Depressive symptoms at BL*Group		-.05	.05	-.15 to .05	.36
Traumatic experiences					
Lack of food or water	1439				
Group		-1.85	1.06	-3.94 to .23	.08
Lack of food or water*Group		.31	.66	-.98 to 1.61	.63
No Medical Access	1439				
Group		-1.57	.94	-3.42 to .26	.09
No Medical Access*Group		-.15	.57	-1.28 to .97	.78
Lack of Shelter	1437				
Group		-1.74	1.04	-3.78 to .30	.09
Lack of Shelter*Group		.19	.65	-1.11 to 1.46	.77
Imprisonment	1437				
Group		-1.78	.93	-3.60 to .04	.05
Imprisonment *Group		.42	.69	-.92 to 1.78	.54
Serious Injury	1437				
Group		-2.11	.96	-3.99 to -.23	.02
Serious Injury*Group		1.30	.60	.13 to 2.46	.03**
Combat	1438				
Group		-2.27	.75	-3.75 to -.79	.003
Combat*Group		1.21	.71	-.18 to 2.60	.09
Rape or Sexual Abuse	1437				
Group		-1.52	.87	-3.23 to .19	.08
Rabe or Sexual Abuse*Group		-.90	.79	-2.45 to .65	.25
Close to death	1437				
Group		-1.36	.95	-3.22 to .50	.15
Close to death*Group		-.64	.55	-1.72 to .43	.24
Murder	1440				
Group		-1.66	.95	-3.53 to .20	.08
Murder*Group		.01	.55	-1.07 to 1.10	.98
Abduction	1438				

Group		-1.50	.96	-3.38 to .37	.12
Abduction*Group		-.71	.65	-1.99 to .57	.28
Torture	1438				
Group		-1.50	.91	-3.28 to .28	.10
Torture*Group		-.35	.60	-1.54 to .83	.56
Wellbeing	1433				
Group		-2.84	.92	-4.65 to -1.02	.002
Wellbeing*Group		.03	.01	.01 to .05	.01**
Self-identified problems	1290				
Group		-1.63	1.16	-3.90 to .63	.16
Self-identified problems*Group		-.013	.06	-.13 to .11	.83
Functioning	1393				
Group		-.95	.75	-2.43 to .52	.20
Functioning *Group		-2.45	1.54	-5.50 to .58	.11
Distress levels	1434				
Group		-1.69	.85	-3.35 to .03	.05
Distress levels*Group		.10	.27	-.43 to .63	.71

Abbreviations: BL: Baselin; CI: Confidence Intervals; Coef: Coefficient; Nobs: Number of observations; p: p-value
